# Snežna jama (Slovenia): Interdisciplinary dating of cave sediments and implication for landscape evolution

**DOI:** 10.1016/j.geomorph.2014.12.034

**Published:** 2015-10-15

**Authors:** Philipp Häuselmann, Andrej Mihevc, Petr Pruner, Ivan Horáček, Stanislav Čermák, Helena Hercman, Diana Sahy, Markus Fiebig, Nadja Zupan Hajna, Pavel Bosák

**Affiliations:** a Swiss Institute for Speleology and Karst Studies SISKA, c.p. 818, 2301 La Chaux-de-Fonds, Switzerland; b Karst Research Institute, Scientific Research Centre, Slovenian Academy of Sciences and Arts, Titov trg 2, 6230 Postojna, Slovenia; c Institute of Geology of the Academy of Sciences of the Czech Republic, v.v.i., Rozvojová 269, 165 00 Praha 6, Czech Republic; d Department of Zoology, Faculty of Science, Charles University, Viničná 4, 128 43 Praha 2, Czech Republic; e Institute of Geological Sciences, Polish Academy of Sciences, Twarda 51/55, 00-818 Warszawa, Poland; f Institute of Applied Geology, Department of Civil Engineering and Natural Hazards, University of Natural Resources and Applied Life Sciences, Peter Jordan Strasse 70, 1190 Vienna, Austria

**Keywords:** Speleogenesis, Cave sediment dating, Slovenia, Periadriatic fault, Dating methods

## Abstract

Caves are important markers of surface evolution, since they are, as a general rule, linked with ancient valley bottoms by their springs. However, caves can only be dated indirectly by means of the sediments they contain. If the sediment is older than common dating methods, one has to use multiple dating approaches in order to get meaningful results. U/Th dating, palaeomagnetic analysis of flowstone and sediment profiles, cosmogenic dating of quartz pebbles, and mammalian dating allowed a robust estimate of speleogenesis, sediment deposition, climatic change at the surface, and uplift history on the Periadriatic fault line during the Plio-Pleistocene. Our dates indicate that Snežna jama was formed in the (Upper) Miocene, received its sedimentary deposits during the Pliocene in a rather low-lying, hilly landscape, and became inactive due to uplift along the Periadriatic and Sava faults and climatic changes at the beginning of the Quaternary. Although it is only a single cave, the information contained within it makes it an important site of the Southern Alps.

## Highlights

• We present a comprehensive dating approach to sediments of a Slovenian cave.
• We interpret the results in the light of climatic change and tectonic uplift.
• We show that collaborative work on one single cave can yield results of broader interest.

## 1 Introduction

The Raduha Massif in the Kamnik Alps, Slovenia, lies close to the Periadriatic fault. This important tectonic line caused dextral strike–slip faulting and differential uplift (Fodor et al., 1998). In order to reconstruct the valley deepening processes in time, and to quantify uplift activity along the fault, paleoelevation markers are needed. Since caves are usually tied to valley bottoms where their springs are situated, (sub)horizontal (epi)phreatic caves are good markers of relative erosional stillstand or of erosional events (Ford and Williams, 2007; Häuselmann et al., 2007; Wagner et al., 2010). The Raduha Massif contains such a cave, Snežna jama (Ice Cave), at an altitude of 1500 m a.s.l., almost 1000 m above the present-day baselevel. There is no indication of vadose/phreatic transition in Snežna jama, so the exact position of the waterlevel at the time of cave formation cannot be ascertained. Although horizontality is no proof in itself for a position close to the baselevel, speleogenetic research shows that large subhorizontal caves are most of the time closely related to the baselevel (Klimchouk et al., 2000). This indicates strong valley incision and/or uplift since cave formation and has the potential to provide information about landscape evolution in the area.

Caves contain, generally, sediments that are comparatively easy to date with different methods. In the present case, Snežna jama presents more difficulties: U/Th dating yielded age estimates older than the upper limit of the method (Bosák et al., 2002), and paleomagnetism only yields relative ages. To resolve the question of the age of Snežna jama, we had to revert therefore to a combination of absolute and relative methods in order to get an accurate and robust age estimate. In this article, we present the methods used and their results, and we interpret the cave's genesis in relation to activity phases of the Periadriatic fault.

## 2 Situation

**Fig. 1:**
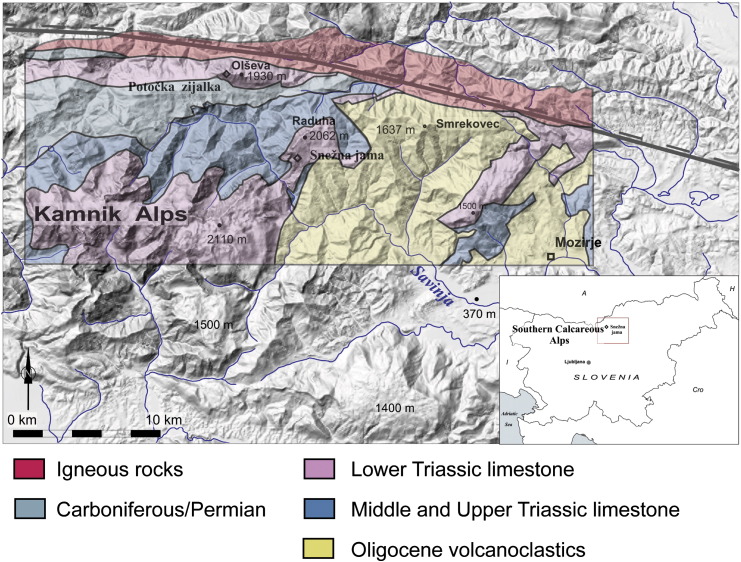
Location of the area in Slovenia and the Southern Calcareous Alps, based on a digital elevation model. The Periadriatic line is shown on the upper part of the figure. A simplified geological map is added.

The Kamnik Alps are a part of Southern Calcareous Alps. They are built of thrust sheets of Triassic sediments (carbonates, sandstones, marls) and volcanics (keratophyre, porphyre and diabase). The Raduha Massif is an eastern carbonate promontory, consisting of massive and thick-bedded Upper Triassic limestone (Fig. 1). Deeply entrenched valleys separate it from other ridges. Strong tectonic activity along the Periadriatic fault, north of the Raduha Massif, caused dextral slip faulting, folding and strata tilting as well as general and differential uplift (Fodor et al., 1998).

**Fig. 2:**
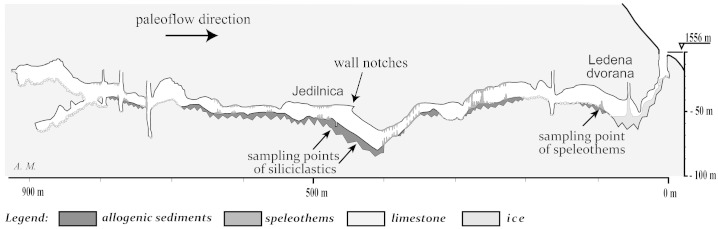
Schematic cross-section along Snežna jama. The sampling points and the location of the wall notches are indicated.

**Fig. 3:**
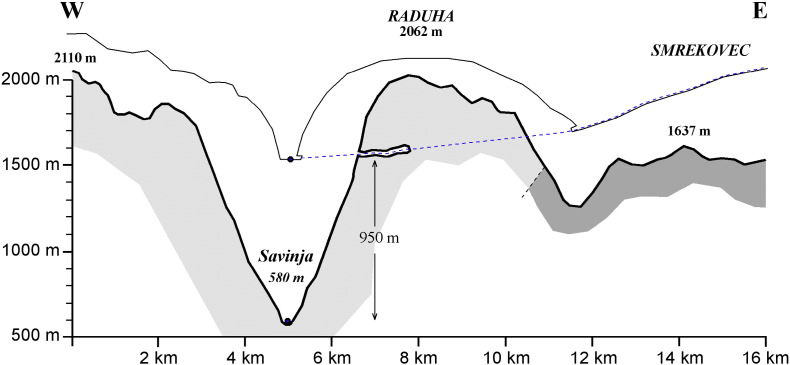
Schematic cross-section showing the position of Snežna jama relative to Savinja river and Smrekovec, the source area of fluvial vulcanoclastic sediments. In fine lines, a schematic sketch of the paleolandscape at the time the cave was active and filled with vulcanoclastic sediments.

Several caves, mostly deep shafts, are known in the Raduha Massif. Snežna jama is the most extensive (Fig. 2). The cave is 1600 m long, the main passage of which consists of a large, mostly horizontal, gallery at about 1500 m a.s.l., which is penetrated by four large shafts (Naraglav and Ramšak, 1990). A collapse terminates the cave close to the vertical northern edge of the Raduha Massif. The main gallery of the cave contains up to 20 m of allochthonous volcaniclastic sediments. Their origin is Smrekovec Mountain east of Raduha. At present, the contact of volcaniclastic rocks with limestones lies 1 km east of the cave, but 350 m below it (Fig. 3). Large flowstone masses were deposited over the older sediments. There is no deposition of flowstone in the present climatic conditions. The waters that infiltrate today into Raduha karst reappear in springs in the Savinja Valley at about 580 m a.s.l.

## 3 Sediment profiles

### 3.1 *Ledena dvorana* (speleothems)

**Fig. 4:**
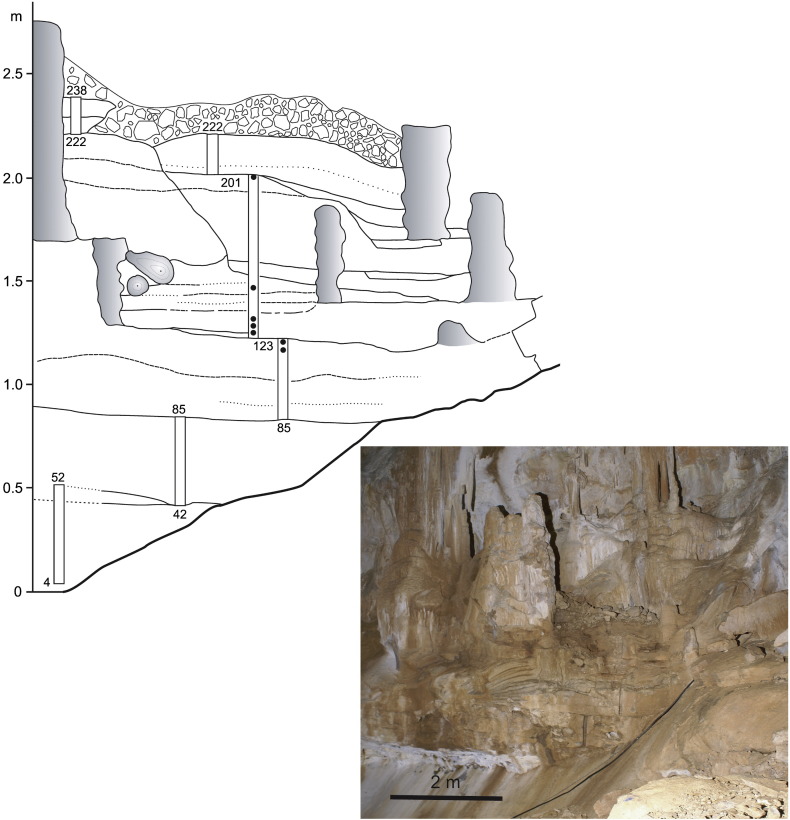
Sketch and photo of the speleothem profile in Ledena dvorana. Rectangular slots indicate sampled parts for palaeomagnetic analyses, and black dots indicate the samples for U/Th. Stalagmites are shaded grey. Numbers indicate elevation above bottom of the profile. Photo A. Mihevc.

The *speleothem profile* (2.4 m of flowstone with only one small intercalation of clayey sand) is situated in the inner part of “Ledena dvorana” (Ice Hall), about 90 m from the entrance to the cave and at a depth of about 45 m. The profile was sampled in 5 separated segments continuously by mostly overlapping trenches cut by circular saw (Fig. 4). The complex sequence of flowstones contains numerous hiatuses; there are six principal flowstone layers with a total thickness of 2.4 m. The lower part of the profile contains abundant terrigeneous components (most probably clay of terra-rossa type). Stalagmites developed in several of the older periods were completely buried by nearly horizontal younger sequences of flowstone. Some stalagmites were buried even after they broke. The lower part of the profile (0–85 cm) is composed of mostly reddish brown to brownish red, sometime light brown, flowstone with some grey bands and reddish brown lamination. It is mostly fine-crystalline and often fenestral (porous to vuggy). Porous bands alternate with massive beds in some sections of the profile. Ferestral structures are coated by finely crystallized and 1–2 mm thick palisadic calcite. The remaining profile is composed of light-coloured flowstones (beige, light ochre, light grey, honey), laminated to banded, partly re-crystallized with bands composed of columnar calcite crystals. Regular alternation of laminated bands, bands with columnar structure, and highly porous bands occurs in places. The porous bands resemble lithified moonmilk layers. Some layers are corroded, especially in the upper part of the profile, and vugs are coated or filled with fine hedgehog-like wall coatings or hedgehog crystal aggregates. Some bands have a chalky appearance and others are pseudo-oolitic at their bases. One thin intercalation of light brown, fine-grained clayey-silty sand was detected at 145 cm.

### 3.2 *Jedilnica* (siliciclastics)

**Fig. 5:**
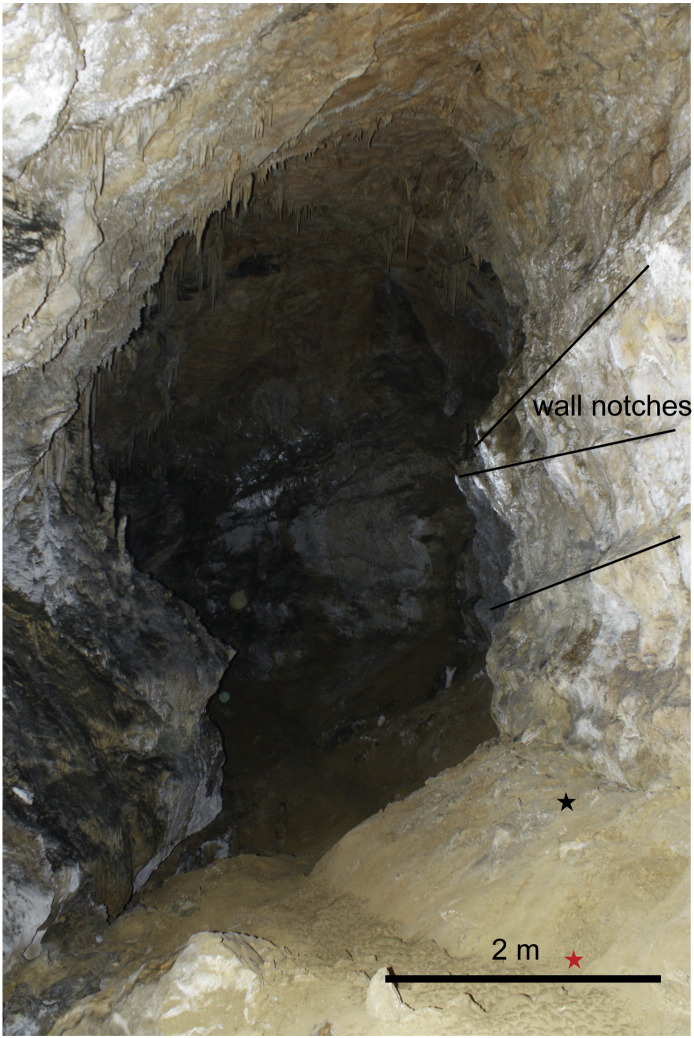
Photo of the passage just downstream of Jedilnica. The passage and the wall notches at the right side are inclined at ca. 20° here. The lower profile (Fig. 7) is situated on the right wall in the middle ground, the situation of the consmogenic samples is indicated by the red star, and the bat nests were found below 5–6 cm of clay at the location indicated by the black star. View is towards the exit of the cave. Photo A. Mihevc.

The siliciclastic sediment profiles are situated about 460 m from the entrance in a place called “Jedilnica” (Dining Room; Figs. 2 and 5), where the main cave passage is 8 to 15 m wide and about 15 m high. The present passage bottom is on top of the sedimentary fill. The passage rises 20 m towards NW in the distance of 50 m. Gravel interbeds occur in the lower part of the slope, while finer siliciclastic sediments prevail in its upper part.

**Fig. 6:**
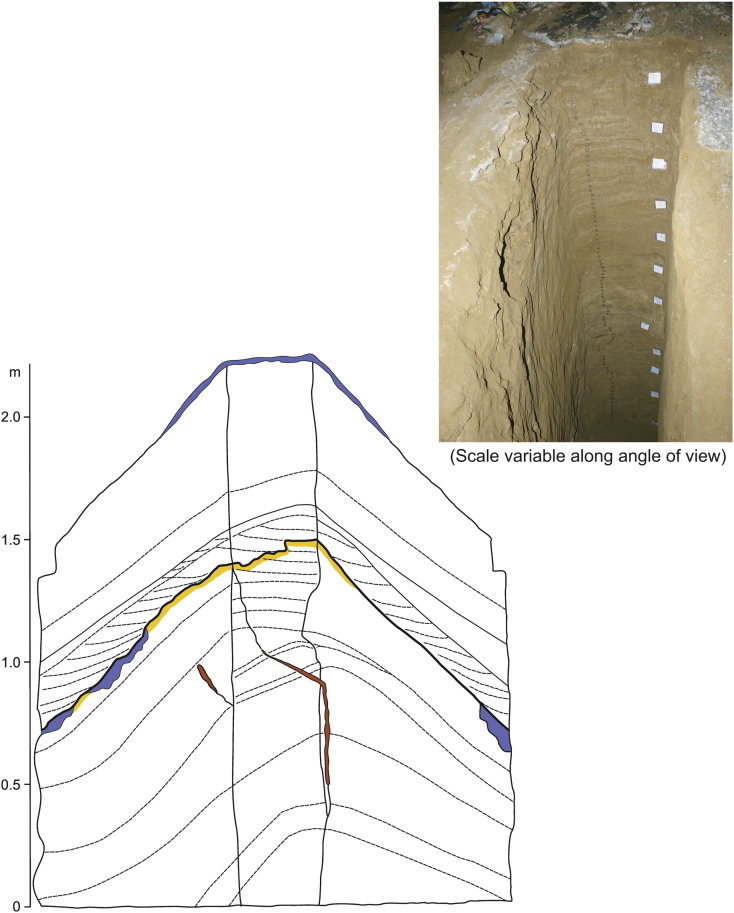
Sketch and photo of the upper profile in Jedilnica, not the whole profile (3.6 m) shown. Sketch: The sketch is halfway 3D (left side, backside, and right side drawn), yellow: weathered layer below the erosional unconformity; red: fissure fill by material from above the erosion line; blue: calcite cementation and/or flowstone; dotted lines: bedding and cross-bedding planes, only most important ones shown. Photo: Sampling boxes for paleomagnetic analysis are visible. The white labels on the right side are 30 cm apart and indicate places of samples for paleontological analysis. Photo A. Mihevc.

The *upper profile* was excavated near the top of the slope. Initial excavations with a 4.8 m hand drill did not reach the bottom of the sediment fill. A 4.3 m deep pit, excavated in two steps was dug to facilitate sampling. Samples for palaeontological analysis were taken in intervals of 30 cm, 20 to 25 kg each. In total, 89 samples for palaeomagnetic analyses were taken as well (Fig. 6), except the topmost 26 cm which is composed of disturbed sediments and covered by porous thin flowstone. The excavated profile consists of two distinct sequences separated by an unconformity. Traces of weathering up to a depth of 5.5 cm below the unconformity are accompanied by local carbonate cementation. The sediment profile generally consists of 2 to 4 cm thick layers of rhythmically arranged clays and silts with medium- to coarse-grained sandy admixture representing weathered (bentonitized) volcaniclastics. Sediments below the unconformity are generally finer-grained, ochre to beige, with brown, violet and reddish brown laminae and bands in the upper part. Convolute/disturbed lamination occurs only locally. The basal 60 cm are of sandy appearance with clayey layers arranged in coarsening-upward cycles. There are many fractures below the unconformity filled with sediments from the upper sequence as result of slope movements during the deposition break. Sediments above the hiatus with fossil weathering are generally more coarse-grained, with distinct clasts (deeply weathered lapilli and pumices) and local small elongated varnished pebbles. The color is ochre, with brown and variocolored laminas and bands in the upper part of the sequence. The sediments are laminated to banded, and some laminae/bands are boudined/disturbed; cross-bedded sets occur only above the sequence base. The content of clay component increases in the upper parts of rhythms (flyschoid style) in both sequences. The stratal dip is uniform from 38 to 42°. The clay fraction is composed of 14-Å-minerals (smectite, chlorite), illite, kaolinite, quartz, plagioclase and calcite, that were determined by XRD.

**Fig. 7:**
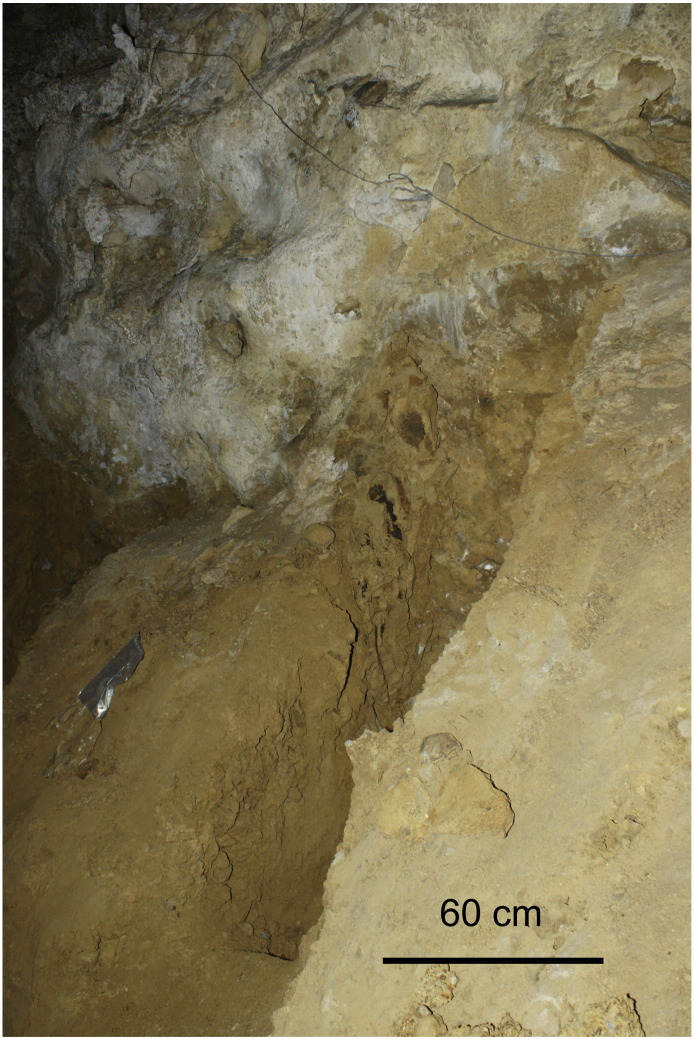
Photo of the lower profile in Jedilnica. Photo A. Mihevc.

The *lower profile* (natural outcrop) at the base of the slope is 2 m high and attached to the cave walls (Fig. 7). The bottom is composed of mud-supported gravel derived from re-deposited volcaniclastics with individual limestone pebbles. The pebbles (trachytes?) are well-rounded and varnished. The upper part is composed of ochre clays with two sets of distinct fissures. The sediments are separated from the cave walls by structures resembling telescoping neptunian dykes filled with subparallel injections of clayey and silty mud with sandy admixture and clay clasts. The dykes are up to 1 m thick, pinching down. This part of the profile was not sampled for paleomagnetics. Such structures were not detected in the upper pit; they cannot influence therefore the paleomagnetic results. The limestone walls are covered by films composed of Mn/Fe compounds. *Liesegang* features were detected as well in sediments. Nests of bat bones were discovered near and at the top in places (Fig. 5).

## 4 Dating methods and results

### 4.1 U/Th

U/Th dating (α-spectrometry method) was carried out in the Geochronology Laboratory of the Institute of Geological Sciences, Polish Academy of Sciences in Warsaw. Samples were taken at 117, 121, 125, 129, 131 and 147 cm, counted from below. Samples 121 and 147 did not yield results because of detrital contamination. Results are on Table 1. Standard chemical procedures for uranium and thorium separation from carbonate samples were used (Ivanovich and Harmon, 1992). Activity measurements were performed using OCTETE PC made by EG&G ORTEC. Spectra analyses and age calculations have been done using “URANOTHOR 2.5” software, which is standard software used in the U-Series Laboratory in Warsaw (Gorka and Hercman, 2002). The quoted errors are one standard deviation.

All samples are characterized by low U content. As a result, the accuracy of analyses is rather low. In addition, there was significant contamination by detrital Th in samples W809 and W806 (^230^Th/^232^Th < 20). ^230^Th/^234^U activity ratios are in equilibrium within the error range (1 standard deviation). ^234^U/^238^U activity ratios, except the samples with significant detrital contamination, are also in equilibrium within the error range. This suggests ages > 1.2 Ma. This conclusion must be accepted with caution, however, because of the low accuracy of activity ratios.

### 4.2 Cosmogenic dating

#### 4.2.1 Sampling

Allochthonous pebbles mostly composed of andesite tuffs and tuffites prevail, while poorly-rounded autochthonous limestone pebbles are less frequent. Quartz pebbles and coarse quartz sand are present in very minor quantities; therefore, a lot of sample had to be carried out of the cave in order to get sufficient quartz for dating. The pebbles were taken from the lower profile, that is, from the natural outcrop.

#### 4.2.2 Method

The burial age method involves the measurement of two isotopes (^26^Al and ^10^Be) that are produced by cosmic radiation in quartz near the surface prior to burial. ^26^Al and ^10^Be accumulate at a ratio of about 6.8:1 in quartz grains with a rate of a few atoms per gram of quartz per year. Sufficiently deep burial (more than 10 m) of such quartz-rich sediment in a cave assures shielding from further cosmic rays. After burial the ^26^Al and ^10^Be concentrations in the sample are only affected by their relative decay resulting in a decrease in the ^26^Al/^10^Be ratio. The measured ratio can be used to derive a burial age (Gosse and Phillips, 2001; Granger and Muzikar, 2001). The current upper limit for measurement of the ^26^Al and ^10^Be isotope pair is around 5 my. A prerequisite of the burial dating technique is that samples have been exposed long enough to cosmic rays and accumulated sufficient cosmogenic nuclides prior to burial. Unfortunately this cannot be determined a priori in the field.

The isotope concentrations can also be used to infer paleo-erosion rates of the source area prior to burial of the clasts. This is accomplished by backward modeling the quantity of nuclides present prior to the burial coupled with local production rate estimates. The pre-burial ^26^Al/^10^Be ratio (~ 6.8:1) is basically not influenced by production rate and thus elevation (Nishiizumi et al., 1989; Stock et al., 2005) and therefore burial ages remain unaffected by altitude changes in the source area. However, the pre-burial erosion rates are based on measured isotope concentrations and elevation dependant production rates. They are therefore only approximate.

In the laboratory, about 100 g of quartz was extracted and purified from bulk samples by magnetic and density separation and selective chemical dissolution. Quartz was dissolved in a 5:1 solution of concentrated HF and HNO_3_ and spiked with about 0.35 mg ^9^Be. Al and Be were separated and purified by ion chromatography and selective precipitation. Precipitates were oxidized and mixed with metal powder for accelerator mass spectrometry (AMS). ^10^Be/^9^Be and ^26^Al/^27^Al nuclide ratios in the sample and procedural blanks were measured at Purdue University in West Lafayette (USA). Stable aluminium concentrations were determined by ICP-OES. The stated errors are 1σ calculated from AMS and ICP-OES uncertainties.

#### 4.2.3 Results

The calculated burial age for the sample SN yielded an age of 3.57 ± 1.23 Ma (Table 2).

The error of 1.23 Ma is large and due to the scarcity of Al atoms still present in the sample. Most probably, the time between erosion and burial of the sample was too short, so that the sample did not accumulate a large number of nuclides. Together with a comparatively long burial, reducing the nuclides, the sample was close to the measuring limits. However, both Al and Be isotopes are low and thus reflect a rather high burial age, and we can assume that the calculated age (with error) is most probably correct.

Approximative erosion rates were calculated with an elevation of the catchment area of 600 m a.s.l. The resulting erosion rate of 28 m/Ma is smaller than its error (77 m/Ma), evidencing the difficulty of correct erosion rate estimates.

Basic data is given in the supplementary data section.

### 4.3 Paleomagnetism

The speleothem profile in Sněžna jama was the first one where the high-resolution sampling approach was used (Bosák et al., 2002, 2003). Profiles were sampled in two places: speleothems from Ledena dvorana (100 samples) and siliciclastics from the excavated pit at Jedilnica (121 samples). Here we present a concise summary of the paleomagnetic data from the Ledena dvorana speleothem published by Zupan Hajna et al. (2008) and compare it with the new sediment profile in Jedilnica.

#### 4.3.1 Methods

Palaeomagnetic analyses were completed in the Laboratory of Palaeomagnetism, Institute of Geology, Academy of Sciences of the Czech Republic in Praha–Průhonice. All field hand specimens were oriented in situ. Unconsolidated sediments were sampled in small non-magnetic plastic cubes (20 × 20 × 20 mm, with a volume of ~ 8 cm^3^). Samples from consolidated rocks and speleothems were collected from the profile in large pieces, which were cut in laboratory to cubes of 20 × 20 × 20 mm. Samples were demagnetized by alternating field (AF; all samples) and or/thermal demagnetization (TD; consolidated samples). The Schonstedt GSD-1 or LDA-3 apparatus was employed for the AF demagnetization. The alternating field (AF) demagnetization was carried out up to a field of 100 mT in 12–16 steps. A MAVACS apparatus (Příhoda et al., 1989) was used for the thermal demagnetization (TD). The natural remanent magnetization (NRM) was measured on JR-6A spinner magnetometers (Jelínek, 1966) and/or a 2G Superconducting Rock Magnetometer with incorporated AF unit. The NRM of specimens is identified by the symbol *J*_n_. The magnetic susceptibility (MS) values were measured on the KLY-2 (or KLY-3) kappa-bridges and a KLF-4A Automatic Magnetic Susceptibility Meter (Jelínek, 1966, 1973).

The characteristic remanent magnetization (ChRM) of each sample was determined by subjecting its demagnetization results to the principle component analysis technique of Kirschvink (1980). Except for samples with very low intensity, the maximum angular deviation (MAD) values are generally lower than 10°; therefore the paleomagnetic directions are well determined. Samples with values (MAD) greater than 10° are interpreted with transient polarity. The ChRM directions were clearly dominated by normal polarity (positive inclinations) and/or reverse polarity (negative inclinations).

To test the possible influence of phase changes of magnetic minerals during laboratory TD processing, diagrams of k_t_/k_n_ values vs. laboratory thermal demagnetizing field t (°C) were also constructed for the samples. In the first stage of laboratory studies, pilot samples were subjected to the analysis of the IRM acquisition and AF demagnetization curves with the aim to establish magnetic hardness of the magnetically active minerals contained in the sediments. Six pilot samples were subjected to processing of the IRM acquisition up to the field of 1 T and to subsequent AF demagnetization. The IRM acquisition showed that all samples reached magnetic saturation by 80 to 160 mT. Backfield demagnetization of the saturation IRM revealed a coercivity of remanence between 10 and 20 mT in general. *A-components* of remanence are mostly of viscous or chemoremanent (weathering) origin; they can be removed by AF (TD) demagnetization with an intensity of 1–5 mT (100 °C). The *B-low-field (low-temperature) component* is secondary but shows harder magnetic properties and can be demagnetized in the AF and or TD (5–15 mT, 100–350 °C). The characteristic *C-high-field (high-temperature) component* is stable and can be isolated in the AF and or TD (ca 15–80 mT, 350–540 °C). Fisher (1953) statistics were employed for the calculation of mean directions of the natural remanent magnetization (NRM) components derived by the multi-component analysis.

**Fig. 8:**
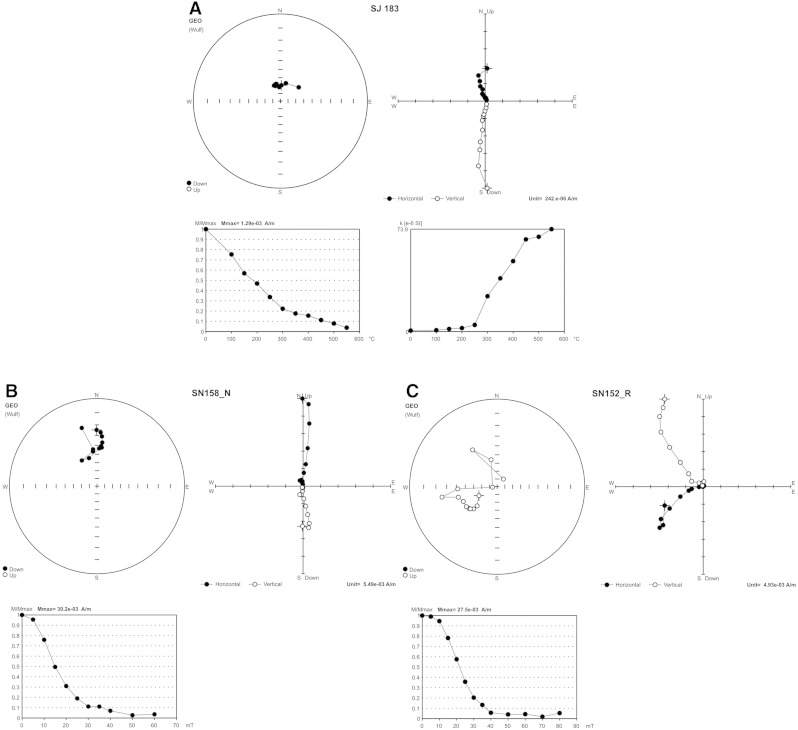
Results of sample demagnetization from Snežna jama. A) Ledena dvorana, TD demagnetization: SJ 183 (N polarity); B) Jedilnica, AF demagnetization SN 158 (N polarity), and C) SN 152 (R polarity). A stereographic projection of the NRM of a sample in the natural state (cross-section) and after the progressive TD and/or AF demagnetization. Zijderveld diagram—solid circles represent projection on the horizontal plane (XY), open circles represent projections on the north–south vertical plane (XZ). A graph of normalized values of the remanent magnetic moments versus thermal demagnetizing fields; M—modulus of the remanent magnetic moment of a sample subjected to the TD and/or AF demagnetization. A graph of the normalized values of volume MS versus TD fields; k—value of volume MS of a sample subjected to the TD.

As a complementary technique to magnetostratigraphy, the measurements of the anisotropy of the low-field magnetic susceptibility (AMS) were performed throughout the section. The AMS was measured with an Agico KLY-4S Kappabridge with an alternating field intensity of 300 A/m and an operating frequency of 875 Hz. The AMS of any rock is dependent on the intrinsic magnetic susceptibility, volume fraction, and degree of preferred orientation of the individual rock-constituent minerals (Jelínek, 1981). Results of MAVACS-operated TD procedures are exemplified in Fig. 8A. They refer to one flowstone sample with normal (N) palaeomagnetic directions (sample SJ 183). Average unblocking temperatures of 580 °C point to magnetite as the principal carrier of magnetization. Examples of the AF demagnetization are illustrated in Fig. 8B, C for a sample with N palaeomagnetic directions (sample SN 158_N) and a sample with R palaeomagnetic directions (SN 152_R). A detailed description of the applied palaeomagnetic method, data interpretation and correlations are summarized by Zupan Hajna et al. (2008, 2010), including a detailed summary of the speleothem profile in Ledena dvorana (p. 212–219).

As mentioned already by Bosák et al. (2000), the sedimentary fills of a number of studied profiles in cave sediments were separated into individual sequences and cycles, mostly of fluvial or ponding nature, because they were divided by breaks in deposition (unconformities). Unconformities and/or intercalated precipitates indicate the highly complicated deposition dynamics in a cave system which can be completely filled and emptied several times. Some of the unconformities were expressed by post-depositional changes, erosion and/or precipitation features. These events can result not only from fluvial-type erosion, but also from oscillations of the karst water level within caves. Unconformities within sedimentary profiles can hide substantial amounts of geological time (Bosák et al., 2000, 2003). Subchron geometry and biostratigraphic data in some Slovene caves helped to estimate that the duration of individual breaks was usually about 150–250 ka and less, but sometimes substantially more, up to 0.7–1 Ma (Zupan Hajna et al., 2008). The geometry of the magnetostratigraphic log in most studied caves, not only in Slovenia, was disturbed by breaks in deposition; therefore magnetozones can start and/or terminate on such unconformities. This is comparable with the situation detected on Quaternary carbonate platforms, where short depositional events are interrupted by prolonged hiatuses (McNeil et al., 1988, 1998). The general character of cave depositional environments with their numbers of post-depositional changes, hiatuses, reworking and re-deposition does not allow precise calculation of the temporal duration of individual interpreted magnetozones. All these factors contribute to the fact that exact calibration of the geometric characteristics of the magnetostratigraphic logs with the GPTS cannot be attained at all or only with problems, if it is not adjusted using results of other dating and geomorphic methods (Bosák et al., 2000, 2003; Zupan Hajna et al., 2008).

#### 4.3.2 Results

##### 4.3.2.1 Ledena dvorana

**Fig. 9:**
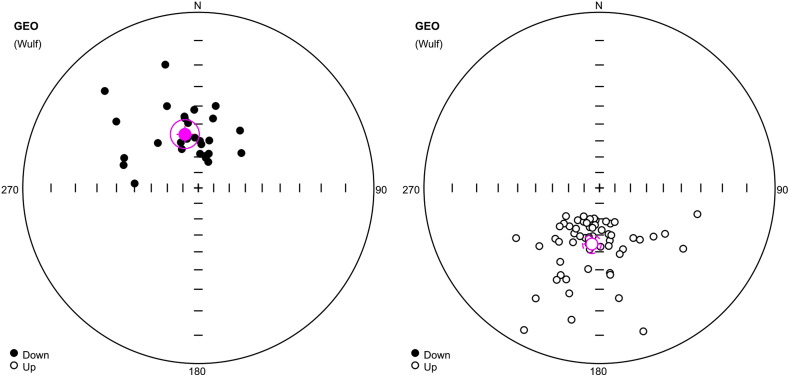
Directions of C-components of remanence with N (left) and R (right) polarity from speleothems, Ledena dvorana, Snežna jama. Stereographic projection, open (full) small circles represent projection onto the lower (upper) hemisphere. The mean direction calculated according to Fisher (1953) is marked by a crossed circle; the confidence circle at the 95% probability level is circumscribed around the mean direction.

The mean *J*_n_ and *k*_n_ moduli values from four segments in the profile (total of 100 samples) are documented in Table 3. The Fisher distribution (1953) displays two well-defined sets of samples with normal (N) and reverse (R) polarities (Fig. 9; Table 5). The top of the profile displays a N1 magnetozone (227–238 cm, Fig. 11). Two narrow N2 and N3 magnetozones (201–205 cm and 179–182 cm) are detected in two narrow R1 and R2 magnetozones (206–226 cm and 183–200 cm) and the long R3 zone at 115–225 cm). The middle part of the profile displays two narrow N4 and N5 magnetozones (from 100 to 113 cm and 72 to 88 cm) and one R4 polarity zone (88–99 cm). The lower parts of the profile from 7 to 70 cm display two N magnetozones (38–45 cm and 7–20 cm) and two R magnetozones (44–68 cm and 20–38 cm; Fig. 11).

##### 4.3.2.2 Jedilnica

**Fig. 10:**
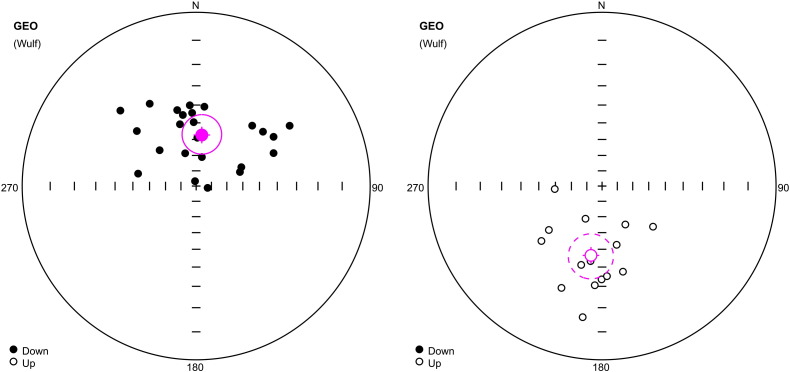
Directions of C-components of remanence with N (left) and R (right) polarity, siliciclastics at Jedilnica, Snežna jama. Explanations: see Fig. 9.

**Fig. 11:**
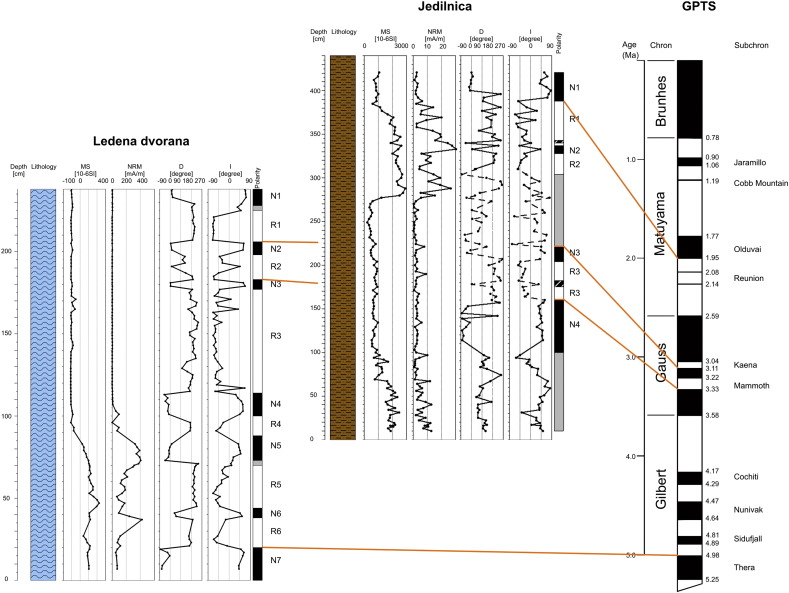
Correlations of magnetostratigraphy interpretation of speleothem profile in Ledena dvorana (modified from Bosák et al., 2002), siliciclastics in Jedilnica and the GPTS (modified from Cande and Kent, 1995). Compare with Table 6. Explanations: profiles of basic magnetic and magnetostratigraphic parameters: blue—speleothems; brown—siliciclastics; magnetostratigraphy columns: white—R polarity; black—N polarity; grey—transient polarity.

The mean *J*_n_ and *k*_n_ moduli values in four segments of the profile are documented in Table 4. Only 28 samples from 121 indicate clear N or R polarity, 90 samples are rather of N or R polarity, and the rest show unclear polarity. The classical “fold test” just to compare the statistic parameters α_95_ (semi-vertical angle of the cone of confidence) calculated according to Fisher (1953) at the 95% probability level and k (precision parameter) could not be applied. Results of mean direction of the C-component of samples corrected and uncorrected for the bedding tilt (in situ directions) are summarized in Table 5. The mean palaeomagnetic directions of the N polarized C-components for uncorrected samples (in situ directions) are D = 6.26°, I = 57.07°, α_95_ = 10.7° and those for tilt-corrected samples are D = 49.55°, I = 50.7°, α_95_ = 10.8°. R polarized C-components for uncorrected samples (in situ directions) are D = 189.06°, I = − 46.01°, α_95_ = 11.4°; for corrected samples they are D = 227.16°, I = − 35.9°, α_95_ = 12.81°. The estimate of the precision parameter κ for the corrected directions is highly formal. This experiment shows the negative fold test. We see that α_95_ are smaller and k larger for samples “in situ” than for corrected samples. For this reason, we used “in situ” palaeomagnetic direction for interpretation of data. Two intervals of deviating declinations and inclinations are documented within the Jedilnica profile. Values of MAD are greater than 10 and samples are interpreted with transient (N–R) polarity. The Fisher distribution (1953) displays two well-defined sets of samples with N and R polarities (Fig. 10, Table 5). The top of the profile displays a N1 magnetozone (69–98 cm, Fig. 11). The narrow N2 magnetozone (145–161 cm) is detected between two R-polarized R1 and R2 zones at 100–143 and 162–184 cm. The middle part of the profile display a transient polarity zone, narrow N3 polarity (from 268 to 285 cm) and one R3 polarity zone (289–328 cm). The lower part of the profile displays a N4 polarity zone (333–390 cm) and terminates by a transient polarity zone (Fig. 11).

### 4.4 Mammal dating

#### 4.4.1 Method

16 samples, each containing 20–25 kg of sediment, were taken for palaeontological analyses from the excavated profile at intervals of 30 cm. Small vertebrate fossils were extracted by hand-picking from screen-washed concentrates. Drawing and measurements were taken with the aid of a binocular microscope with camera lucida and ocular micrometer. All measured data are given in millimetres.

#### 4.4.2 Results

The excavated section in **Jedilnica** provided only few fossils, all very fragmentary and poorly preserved. In several layers, the undeterminable teeth fragments of cartilaginous (Chondrichthyes; at least two distinct taxa) and albulid fish were found, obviously re-deposited fossils from Upper Oligocene source rocks. However, sample 11 provided more significant remains, notably small rodent teeth. Besides small fragments of enamel (two of an incisor, one of a wall of a hypsodont molar possibly an arvicolid) which do not allow detailed identification, a well preserved enamel coat of a left third lower molar (m3) belonging to a hypsodont cricetid tentatively attributed to the genus Baranomys was found.

**Fig. 12:**
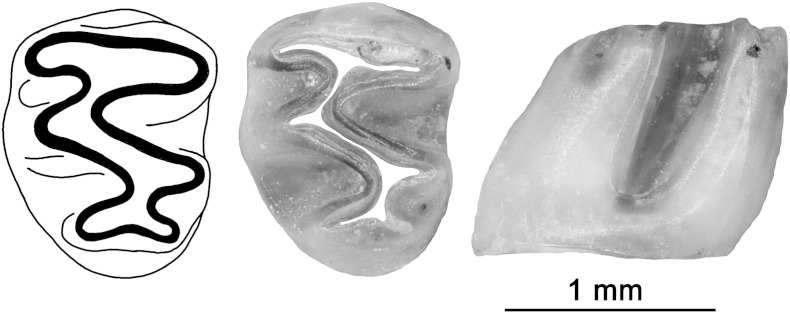
A left third lower molar (m3) of *Baranomys* sp. from excavated Jedilnica section of Snežna jama, sample 11. Occlusal view and buccal views. Scale 1 mm.

The tooth (Fig. 12) belongs to a young individual; it is unworn with well-preserved details of the initial design of the occlusion enabling us to formulate also some comparative notes. It is characterized by a flat occlusal surface with confluent alternating cusps combined with mesodont cricetid design of the crown without any tendency to undulate its basal enamel margin. In all these respect it shows the diagnostic characters of microtoid cricetids, the clade parallel to the earliest radiation of arvicolids appearing in the European fossil record from the latest Miocene to early Pliocene (Fejfar, 1999; Fejfar et al., 2011). In regard to phenotypic variation in that group, the tooth is further characterized by (1) a very small size (L_ocl_ 1.08 mm, L_max_ 1.35 mm, W_ocl_ 0.98 mm, W_max_ 1.04 mm), (2) relatively thick enamel, (3) relatively high degree of hypsodoncy with broad central occlusal plane, (4) deep synclinales and (5) a robust crown base. In regard to the state of these characters in particular genera of the clade (i.e. *Germanomys, Bjornkurtenia, Wartamys, Celadesia* and *Baranomys*) the tooth exhibits the best correspondence to patterns characterizing the genus *Baranomys*, i.e. the youngest and the most common representative of the group and the index genus of the lower Pliocene (MN14–MN16). It is much smaller than corresponding teeth in *Germanomys*, *Bjornkurtenia, Wartamys*, and *Celadesia*, and most of the published data on *Baranomys* s.s. (i.e. *B. loczyi* and *B. longidens*) show somewhat larger size. Nevertheless it falls into the variation range of *B. longidens* reported from MN15 site Węże by Sulimski (1964). Of course, to establish a reliable species identification based on just a single tooth is virtually impossible—therefore we identify the respective specimens as *Baranomys* sp.

**Fig. 13:**
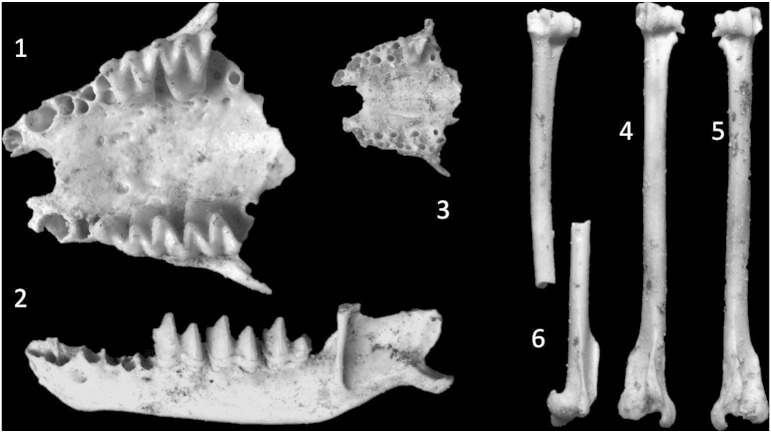
Bat remains from the surface layer of Jedilnica section of Snežna jama. 1—rostrum of *Myotis myotis*, 2—left mandible of *Myotis myotis*, 3—rostrum of *Pipistrellus pipistrellus*, 4,5—humeri of *Pipistrellus pipistrellus*, and 6—humeral fragments of *Myotis* cf. *mystacinus.*

The surface layer at the foot of the sediment slope in **Jedilnica** provided osteological material consisting of ca 25 well preserved fragments, mostly postcranial bones and crania, belonging to 3 individuals of 3 species of bats: *Myotis myotis* (nearly complete rostrum, left mandible), *Pipistrellus* cf. *pipistrellus* (left and right humerus, rostrum) and *Myotis* cf. *mystacinus* (distal and proximal fragments of humerus). All three species (Fig. 13) are common elements of the present bat fauna in the region and most probably were distributed there throughout most of the Quaternary period. The relatively good preservation of fossils excludes post-sedimentary transport. Incomplete patterns of fossilization supports a Holocene or Late Pleistocene age, rather than an older age, which is in agreement with previous finds (Sigé et al., 2003).

### 4.5 X-ray analysis

Powder samples were not specially processed. Separation of the clay fraction was performed in an Andreasen cylinder. The clay fraction was deposited on glass and desiccated for orientation of clay particles. The same specimen mounts have then been saturated with ethylenglycol for 4 h at 80 °C and analyzed again. The glass sample supports have then been heated at 550 °C under ambient atmosphere for 1 h; after cooling the samples they have been scanned under the identical instrumental conditions again.

The X-ray powder patterns have been acquired with a Bruker D8 DISCOVER diffractometer in the Bragg–Brentano θ–2θ reflecting geometry (Institute of Geology AS CR, v. v. i., Praha, Czech Republic). To collect data, a copper radiation has been used. The tube has been operated at 40 kV and 40 mA. The radiation has been monochromatized with a primary asymmetric focusing germanium [(111) cut] monochromator, so the primary beam contained the Kα1 spectral line only. Diffracted radiation has been detected with a position-sensitive linear silicon-strip detector LynxEye. The beam has been constrained with a set of fixed divergence slits and a secondary 2.5° Soller slit.

## 5 Discussion

### 5.1 Magnetostratigraphy

A complex magnetostratigraphic picture was obtained by high-resolution palaeomagnetic analysis both of speleothems (Ledena dvorana) and siliciclastics (pit in Jedilnica). High-resolution sampling contributed to the precision of detection of individual subchron boundaries.

In **Ledena dvorana**, a total of seven N-polarized zones are separated by six R-polarized zones. All samples are older than 350 or 200 ka according to U/Th dating, and the uranium isotopic equilibrium indicates an age above 1.2 Ma (Bosák et al., 2002). It may be supposed that the profile represents a time sequence not younger than the Matuyama chron (1.77 Ma, Olduvai C2n subchron). The profile can be correlated with the Gauss and Gilbert chrons (about 2.6 to 5 Ma; Table 6). The base of the top N1 magnetozone can be identified with 3.04 Ma (C2An.1n subchron; Cande and Kent, 1995) and the top of the N7 with 4.98 Ma (C3n.4n subchron; the base at 5.23 Ma) in the Gauss chron. The growth of speleothems took place over an approximate time span of 1.8 to 2.0 Ma, which gives mean speleothem growth rates of about 1.1 to 1.3 m per 1 Ma.

Rarely, magnetozones terminate and/or start on expressive bedding planes, representing interruption of deposition and thus a hiatus, in flowstone (85, 201 and 222 cm; Fig. 4), but only some of them are expressed by stalagmite growth. Other bedding planes accompanied by growth of columnar stalagmites are situated within the individual magnetozones. The geometry of detected magnetozones, compared with the geomagnetic polarity timescales (GPTS; Fig. 11) is only slightly deformed, which indicates that duration of breaks expressed by bedding planes in deposition was rather short-lasted (Bosák et al., 2002). Stalagmites are buried in horizontally bedded flowstone layers, which fully record the time of stalagmite growth.

The distribution of the NRM and total MS indicates deposition in two distinctly separated sequences (Fig. 11). The lower part of the profile has substantially higher NRM values. It is composed of brown speleothem with high content of terrigeneous-derived material (reworked and re-deposited terra rossa-type of weathering profile). The upper part, above ca 103 cm, composed of light-coloured flowstones, shows negligible NRM values, i.e. weak admixture of clastic material. The MS curve shows a similar trend. The only thin intercalation of light brown, fine-grained clayey-silty sand was detected at 145 cm indicating parallel deposition of siliciclastic deeply within the cave.

The interpretation of the NRM and MS curves is highly problematic without a final stable isotope curve. If the MS record represents the reflection not only of the iron content in the terrigeneous influx to the cave, but also the climatic conditions, then we can expect different palaeo-environmental conditions during the deposition of two segments of the profile (Bosák et al., 2002).

In **Jedilnica pit**, a total of 4 principal N-polarized zones and 3 principal R-polarized zones were interpreted. *Baranomys* fragments in sample No. 11 indicate mammalian zone 15b to MN16a (ca 2.7–3.9 Ma). The location of the sample is within the N-polarized magnetozone which corresponds to C2An.3n subchron (3.33 to 3.588 Ma) as the highest probability correlation (Table 6). According to this interpretation, it may be supposed that the profile represents a time sequence not younger than the Matuyama chron (1.77 Ma, Olduvai C2n subchron). The base of the top N1 magnetozone represents most probably the base of the Olduvai C2n subchron (1.99 Ma). The profile terminates with a segment with transient polarity, which cannot be older than ca 4.91 Ma according to cosmogenic dating. Therefore the profile correlates with the Matuyma, Gauss and Gilbert chrons.

The profile is divided into two distinct parts by the unconformity accompanied by fossil weathering and speleothem precipitation (at 216 cm), which is well-defined both on NRM and MS curves (Fig. 11). The results of subterranean in situ weathering of sedimentary profiles are quite rare not only in Slovenia (Zupan Hajna et al., 2008, 2010), but also elsewhere (Bosák et al., 2003). The weathering crust indicates moist and warmer cave climate than the present one and/or prolonged weathering. According to correlations with the GPTS, it seems that the hiatus lasted for several 100 ka.

Both R and the middle N magnetozone contain one or more short magnetozones of different polarities; they represent short-lived polarity excursions as detected on one sample only. Zones with unclear/transient polarities correspond to (1) the zone of slumping of unconsolidated sediment below the unconformity surface with open cracks filled with sediments from above the unconformity and the overlying cross-bedded set, and (2) coarse-grained composition of the lower part of the profile.

The distribution of the NRM and especially of total MS indicates a main change of both parameters at 275 cm and at ca. 93 cm. The curve pattern below and above 275 cm is highly similar. Relatively high values in the lower part of the respective segment decrease upwards, which is visible especially on the MS curve. It can indicate the change in composition of clastic material, derived from the catchment area and transported into the cave. This means that eroded bedrock in the catchment had different composition and/or external palaeoenvironmental conditions could differ as well. Higher MS values can indicate more weathered source material in the catchment area, the degree of weathering decreased with continued erosion of source volcaniclastic rocks. The jump in the MS values above 275 cm reflects some climate-change event at the surface, which is expressed also in the cave by in situ chemical weathering of deposits during the hiatus. The deposition of speleothems in Ledena dvorana and siliciclastics in Jedilnica was contemporaneous for most of the time (Table 6), which can be indicated also by a thin siliciclastic intercalation in flowstones in Ledena dvorana (at ca. 145 cm). The deposition in Ledena dvorana started between 4.98 and 5.23 Ma and terminated between 3.04 and 2.581 Ma, i.e. earlier than in the Jedilnica (the start not later than 4.91 Ma according to cosmogenic age, and the termination between 1.77 and 1.95 Ma according to the magnetostratigraphic interpretation; Fig. 11). The termination of siliciclastic deposition in Jedilnica also indicates the timing of surface changes in the catchment, possibly due to the abandonment of the cave by the formative stream resulting from more intensive uplift. The duration of deposition is also remarkable in comparison with other Slovenian caves (*cf*. Zupan Hajna et al., 2008, 2010).

The mean palaeomagnetic direction values of magnetozones both from Ledena dvorana and Jedilnica clearly support the correlation of individual zones with the appropriate age as mentioned above (see also Table 6). The difference in declination is 6°, in inclination it is 8°, which is less than α_95_ (semi-vertical angle of the cone). Mean palaeomagnetic directions for Ledena dvorana are D = 350.4°, I = 54.6°, α_95_ = 6.5° and those for Jedilnica are D = 352.2°, I = 50.2°, α_95_ = 8.0°, values were calculated for samples with the N polarity, R-polarized samples were transformed into N-polarized ones.

Values of mean palaeomagnetic directions of each magnetozone in both profiles clearly show the palaeorotation trend. The upper part (N1 Jedilnica to R2 Ledena dvorana) indicates clockwise rotation (9°) and the middle and bottom parts (N3 to N5 Ledena dvorana) continue to a counterclockwise rotation (up to 18°; see Table 6). Palaeomagnetic directions in R5 to N7 (Ledena dvorana) cannot be calculated because of the large α_95_ (semi-vertical angle of the cone; see Fig. 9).

### 5.2 Fossil record and biostratigraphic dating

Morphological specificities of the specimen identified tentatively as *Baranomys* sp. place it in the clade of microtoid cricetids sensu Fejfar (1999) and, more specifically in its Group G in sense of Fejfar et al. (2011), characterized by mesodont molars with prismatic dental pattern (alternating cusps). Most members of this clade appeared within stratigraphic range MN13 to MN14 except for genera *Bjoernkurtenia* and *Baranomys* which appear also in MN15 and survived with *Baranomys loczyi* to MN16 (LAD Rębielice Królewskie—Kowalski, 2001). For the reasons mentioned above the co-identification of our specimen with the ancient genera *Germanomys, Wartamys* or *Celadesia* can be excluded. Although there is a good correspondence with the population of *B. longidens* from MN15 Węże (also in metric characters), neither the species nor even generic (whether *Baranomys* or *Bjoernkurtenia*) identity of the specimen can be established for certain. The tooth belongs to a young individual, it is not abraded and in comparison to the advanced forms of *Baranomys* s. s. it seems to be even more hypsodont (h 1.05 mm), more gracile with relative thin enamel and well-developed posteroconal extension of entoconid and with deep lingual and labial synclinales separating anterolophus. Compared to the genus *Baranomys* (thick enamel, robust design with deep but spatially reduced synclinales) these characters can be viewed as more primitive, closer to a state expected in ancestors of the discussed genus. Based on just a single tooth the actual meaning of these specificities cannot be evaluated, in fact. For the purpose of biostratigraphical dating the details are not essential. Both *Baranomys* or *Bjoernkurtenia* suggest a stratigraphical span from MN14 to early MN16 (2.7–5.4 Ma) with MN 15b–16a (ca 2.7–3.9 Ma) as the highest probability datum (judging by the contribution of these clades to European communities). Taking into account the position of sample 11 within the Jedilnica profile, the biostratigraphic age of *Baranomys* corresponds to N polarized magnetozone C2An.3n, i.e. 3.33 to 3.588 Ma.

## 6 General history of the cave and its environments

### 6.1 Cave genesis and sediment deposition

A rough reconstruction of the cave development can be deduced both from the cave morphology and its fills: although formal proof of vadose/phreatic transitions are missing in the cave, the size and quasi-horizontal nature of the cave (Klimchouk et al., 2000) suggests that it had to form close to the water table, at least in its latest stages of development. It has to be noted here that according to Worthington (1991), the passage size reflects rather the discharge of the stream flowing through the cave than the time the cave was active in its phreatic state. The subterranean flow had a recharge of several cubic metres per second, estimated by passage size times gravel diameter indicating flow velocity (Hjulström, 1939). Both the dimensions and shape of the main cave gallery indicate a stable karst water level for a prolonged period. The speleogenetic activity had to predate (or to be contemporaneous with) the deposition of sediments. Since the first sediments were deposited around 5 Ma, the cave existed at the end of the Miocene. Theoretically, the void may be substantially older. But since the area might have been affected only by the Badenian transgression, but not by the deposition in a Pannonian Lake system (Jelen et al., 2008), we estimate that the most probable age of the cave is upper Miocene.

According to pebble composition, the catchment area was situated to the southeast of the cave, on Upper Oligocene volcanogenic rocks. Fluvial sediments were deposited in the cave by a sinking river, which transported highly weathered volcaniclastics into the cave. Wall notches in the passage at Jedilnica dip parallel to the sediment floor and indicate phreatic conditions (sump) during sediment deposition in this part of the cave. Wall notches (total 4) with an inclination of 21–28° towards the cave entrance occur above and along the slope (excavated profile), while the inclination is only 10° above the lower sediment outcrop. Minor wall notches at the speleothem profile (Ledena dvorana) are horizontal to slightly subhorizontal with inclination towards the cave interior. The sediment fill at Jedilnica was deposited in a sump from mudflows, forming a structure similar to a subaqueous delta. Mudflows were dense with low water saturation, and moving slowly, probably from a ceasing subterranean stream. Following the paleomagnetic results, deposition continued for a long time (ca 4.6 to > 1.95 Ma) with one substantial interruption. A weathering profile developed at that time, and some slope movement registered by fissures and cracks in the lower sequence, filled by sediments of the upper sequence (Fig. 6). Carbonate cementation was due to dripping water from the cave ceiling. Telescoping mud injections along cave walls are due to rapid sediment shrinkage (dewatering, compaction). Fluids flowing along cave walls are responsible for Mn/Fe films and *Liesegang* features.

Fluvial sediments deposited in Snežna jama were in places covered by thick speleothems, indicating a warm, wet climate and a low altitude of the cave (Audra et al., 2006). The deposition of speleothems ceased due to climate change and/or mountain uplift around 2.5 to 3 Ma, at the end of the Pliocene and the beginning of the Quaternary cold periods.

### 6.2 Uplift and activity along the Periadriatic fault

The area was affected by the Oligocene submarine volcanic activity depositing the rocks of Smrekovec area. There is no trace of a possible Badenian transgression, but sediments of that time might have been deposited onto the area (Jelen et al., 2008). Subsequently, movement along the Sava fault to the South, and the Periadriatic fault to the North, uplifted the middle block of the Kamnik Alps above the basins. We interpret that the cave formed at this moment.

Pebbles of Miocene rocks were found in Potočka zijalka Cave at a similar elevation, but about 10 km to the north of Raduha (Fig. 1). It is possible that both caves were formed in the same prolonged stable period during the Upper Miocene and Pliocene. Both discharged to the Savinja River, which had only a slightly entrenched valley at that time (Mihevc, 2001; Mihevc et al., 2013). The prolonged time of sediment deposition, the presence of small pebbles (instead of cobbles or sand), and the size of the passage indicate a rather low, gentle relief and a discharge of several cubic metres of water. The Savinja River currently discharges into the Celje Basin. If this basin had been significantly lower than the river, headward regressive erosion would have modified the cave. We conclude that Celje Basin was not substantially lower than the cave, and that therefore the whole area had to be low-lying. This is supported by the massive speleothems found in the cave. Since the area is very far from the Mediterranean, a possible Messinian influence is unlikely, moreover because no such possible influence had been described elsewhere in the area.

Rapid mountain uplift along both the Periadriatic and the Sava faults then caused surface river entrenchment and cut off the subterranean karst drainage. The drop of the karst water level of about 900 m created conditions favourable for vertical drainage and invasion vadose shafts. The karst drainage was reorganized and the cave finally became dry (relict) and has experienced very little change since.

The evidence from the cave sediment record suggests fluvial activity ceased between 1.95 and 1.77 Ma. This means that the catchment area had been truncated by surface erosion, and this again points to general uplift, causing enhanced erosion of the surface. We therefore can estimate that the Periadriatic and Sava faults were causing active uplift around the Plio-Pleistocene boundary. This is in accordance with Fodor et al. (1998), but they stipulate only that a second phase of dextral transpression occurred from Mio-Pliocene to today. The present activity of the fault is evidenced also by modern GPS data (Vrabec et al., 2004). To date, there is very little other literature about the activity of the faults in the Mio-Pliocene timespan.

## 7 Conclusion

Using a comprehensive suite of dating techniques including paleomagnetism, mammalian, cosmogenic burial, and U/Th dating enabled us to gain an insight into the speleogenesis, sediment deposition, and fossilization of Snežna jama, Slovenia. Comparison of all methods indicates that speleogenesis occurred in the Upper Miocene, since the oldest sediments found in the cave date from the Mio-Pliocene transition. We further evidenced a climatic change more or less coinciding with the beginning of the Quaternary period (cessation of flowstone growth), and the effects of mountain uplift around 2 Ma, cutting the cave from its catchment area and thus fossilizing it. Given all this information, Snežna jama is an important archive for understanding the evolution of the southeastern Alps.

## Figures and Tables

**Table 1:** U/Th dating results using α-spectrometry, Snežna jama.

Sample [cm]	Lab. no.	U conc. [ppm]	^234^U/^238^U	^230^Th/^234^U	^230^Th/^232^Th	Min. age^a^ [ka]	Remarks
117	W 813	0.037 ± 0.019	1.087 ± 0.555	0.976 ± 0.108	23	> 300	
121	W 809	0.052 ± 0.009	1.142 ± 0.190	1.042 ± 0.071	10		Significant detrital contamination
125	W 810	0.057 ± 0.006	1.063 ± 0.106	0.969 ± 0.051	60	> 300	
129	W 814	0.040 ± 0.010	0.994 ± 0.239	0.913 ± 0.104	40	> 200	
131	W 811	0.070 ± 0.010	0.971 ± 0.144	0.987 ± 0.064	72	> 300	
147	W 806	0.031 ± 0.007	1.403 ± 0.323	0.926 ± 0.072	6		Significant detrital contamination

a Minimum age calculated based on activity ratios and their accuracy.

**Table 2:** The measured cosmogenic nuclide values of sample SN and the calculated ages (error 1sigma). Note that the cave altitude was assumed before recent uplift, and the altitude of catchment was an approximation for calculation of inherited erosion rate.

Burial ages								
				^26^Al	^10^Be		Burial age	Inh. erosion
Cave	Sample	Altitude catchment	Altitude in cave	(10^3^ at/g)	(10^3^ at/g)	^26^Al/^10^Be	(Ma)	(m/Ma)
Snezna jama	SN	600	1400	34 ± 29	28 ± 0.45	1.21 ± 1.07	3.57 ± 1.23	28 ± 77

**Table 3:** Mean palaeomagnetic values and standard deviation in Ledena dvorana.

Snežna jama Ledena dvorana	J_n_ [mA m^− 1^]	k_n_ × 10^− 6^ [SI]	Interval [cm]^a^
Mean value	1.318	− 3.75	238–107
Standard deviation	1.740	11.14
Number of samples	61	61
Mean value	62.677	10.08	103–91
Standard deviation	35.513	13.29
Number of samples	5	5
Mean value	241.020	215.38	83–37
Standard deviation	111.792	47.25
Number of samples	24	24
Mean value	79.307	187.95	27–7

a From top to base.

**Table 4:** Mean palaeomagnetic values and standard deviation in Jedilnica.

Snežna jama Jedilnica	J_n_ [mA m^− 1^]	k_n_ × 10^− 6^ [SI]	Interval [cm]^a^
Mean value	2.733	855.30	68–108
Standard deviation	1.356	130.21
Number of samples	10	10
Mean value	13.406	2187.86	112–216
Standard deviation	7.804	448.09
Number of samples	28	28
Mean value	2.517	644.16	220–399
Standard deviation	1.918	189.45
Number of samples	51	51
Mean value	6.016	1698.6	402–483
Standard deviation	3.465	423.9
Number of samples	32	32

a From top to base.

**Table 5:** Mean palaeomagnetic directions, speleothems at Ledena dvorana and siliciclastics at Jedilnica, Snežna jama.

Snežna jama	Polarity	Mean palaeomagnetic directions		α*_95_* [^o^]	k	n
D [^o^]	I [^o^]
Ledena dvorana	N	345.93	55.13	7.94	11.1	28
R	187.7	− 54.27	5.18	11.8	62
Jedilnica (*in situ*)	N	6.26	57.07	10.7	7.13	24
R	189.06	− 46.01	11.4	10.05	15
Jedilnica (corrected)	N	49.55	50.7	10.8	7.01	24
R	227.16	− 35.9	12.81	7.97	15

Note: N—normal polarity, R—reverse polarity; D, I—declination and inclination of the remanent magnetization before (in situ) and after dip correction; α_95_—semi-vertical angle of the cone of confidence calculated according to Fisher (1953) at the 95% probability level; k—precision parameter; n—number of analysed samples.

**Table 6:** Mean palaeomagnetic directions of magnetozones and correlation of profiles.

Age^a^ [ka]	Polarity chron^a^	Ledena dvorana			Jedilnica		
Magneto-zone	D [°]	I [°]]	Magneto-zone	D [°]	I [°]
(1770–) < 1950	C2n				N1	3	53
1950–2140	C2r.1r				R1	181	− 52
2140–2150	C2r.1n				N2	6	45
> 2150(–2581)	C2r.2r				R2	178	− 46
(2581–) < 3040	C2An.1n	N1	9	56	T1	–	–
3040–3110	C2An.1r	R1	186	− 64
3110–3220	C2An.2n	N2	2	59	N3	358	53
3220–3330	C2An.2r	R2	172	− 58	R3	174	− 51
3330–3580	C2An.3n	N3	352	59	N4^b^	346	53
3580–4180	C2Ar	R3	173	− 57	T2	–	–
4180–4290	C3n.1n	N4	342	52
4290–4480	C3n.1r	R4	174	− 47
4480–4620	C3n.2n	N5	346	49
4620–4800	C3n.2r	R5	–	–
4800–4890	C3n.3n	N6	–	–
4890–4980	C3n.3r	R6	–	–
> 4980(–5230)	C3n.4n	N7	–	–			

Explanations: N—normal polarity, R—reverse polarity; T—transient/unclear polarity; D, I—declination and inclination of the remanent magnetization; – —large α_95_—semi-vertical angle of the cone.

a After Cande and Kent (1995).

b Horizon with *Baranomys*.

## Appendix A Supplementary data

Supplementary material: Detailed measured values of ^26^Al and ^10^Be.

### Google Map

The following KML file contains the Google map of the most important areas described in this article.

Map KML file containing the Google map of the most important areas described in this article.
